# Breast cancer microenvironment and obesity: challenges for therapy

**DOI:** 10.1007/s10555-022-10031-9

**Published:** 2022-04-18

**Authors:** Lauren E. Hillers-Ziemer, Genevra Kuziel, Abbey E. Williams, Brittney N. Moore, Lisa M. Arendt

**Affiliations:** 1 Program in Cellular and Molecular Biology, University of Wisconsin-Madison, Madison, WI 53706 USA; 2 Laboratory of Cancer Biology and Genetics, Center for Cancer Research, National Cancer Institute, National Institutes of Health, Bethesda, MD 20892 USA; 3 Program in Cancer Biology, University of Wisconsin-Madison, Madison, WI 53705 USA; 4 Comparative Biomedical Sciences Program, University of Wisconsin-Madison, Madison, WI 53706 USA; 5 Department of Comparative Biosciences, School of Veterinary Medicine, University of Wisconsin-Madison, Madison, WI 53706 USA; 6 School of Veterinary Medicine, University of Wisconsin-Madison, 2015 Linden Dr. Rm 4354A, Madison, WI 53706 USA

**Keywords:** Obesity, Breast cancer, Cancer-associated fibroblasts, Adipocytes, Macrophages, Extracellular matrix

## Abstract

Women with obesity who develop breast cancer have a worsened prognosis with diminished survival rates and increased rates of metastasis. Obesity is also associated with decreased breast cancer response to endocrine and chemotherapeutic treatments. Studies utilizing multiple in vivo models of obesity as well as human breast tumors have enhanced our understanding of how obesity alters the breast tumor microenvironment. Changes in the complement and function of adipocytes, adipose-derived stromal cells, immune cells, and endothelial cells and remodeling of the extracellular matrix all contribute to the rapid growth of breast tumors in the context of obesity. Interactions of these cells enhance secretion of cytokines and adipokines as well as local levels of estrogen within the breast tumor microenvironment that promote resistance to multiple therapies. In this review, we will discuss our current understanding of the impact of obesity on the breast tumor microenvironment, how obesity-induced changes in cellular interactions promote resistance to breast cancer treatments, and areas for development of treatment interventions for breast cancer patients with obesity.

## Introduction

Obesity is a global epidemic, with the World Health Organization estimating that worldwide adult obesity has nearly tripled since 1975 [1, 2]. In the USA, 70% of adults have a body mass index (BMI) classified as overweight or obese [3]. Obesity significantly increases the risk for developing postmenopausal breast cancer [4–6]. On average, women undergo menopause between the ages of 45 and 55 years old, and approximately 80% of women diagnosed with breast cancer are over the age of 50, and the median age at diagnosis is 62 years of age [7, 8]. Postmenopausal women are most frequently diagnosed with breast cancers that express estrogen receptor alpha (ERα) and progesterone receptor (PR), and risk for diagnosis with this type of breast cancer is strongly correlated with increasing BMI [9–12]. Women with obesity are more likely to develop tumors of the luminal B molecular subtype, which are characterized by higher proliferation rates and reduced relapse-free survival [13, 14]. This subtype includes ERα^+^ and PR^−^ tumors, in which 66% of patients fail to respond or develop early resistance to the selective ER modulator, tamoxifen [15, 16]. The association of obesity with other breast cancer subtypes in postmenopausal women is less clear. Epidemiological studies examining the impact of obesity on the incidence of triple-negative breast cancers (TNBCs), which lack expression of ERα, PR, and HER2, have shown conflicting results [11, 12, 17, 18].

In contrast, epidemiological studies suggest that obesity reduces breast cancer risk in the general population of women prior to menopause [5, 6, 12], suggesting a complex interaction between obesity and breast cancer risk. Similarly, while obesity increases the risk of ERα^+^ breast cancer in postmenopausal women, premenopausal women with obesity may have reduced risk for this breast cancer subtype [11, 19, 20]. However, the incidence of TNBC may be enhanced in younger women with obesity [17, 19–21]. Racial differences may also impact breast cancer risk in young women, as elevated BMI is correlated with increased breast cancer risk in premenopausal Asian women [22]. Obesity may also worsen the impact of other underlying risk factors for breast cancer including familial risk factors, leading to elevated breast cancer risk in selected groups of young women with obesity [17, 20, 21, 23, 24].

Regardless of age or menopausal status, breast cancer patients who are obese have a significantly worse overall and breast cancer-specific survival compared to patients with a BMI in the healthy range [9, 25–27]. At the time of diagnosis, breast cancer patients with obesity more frequently present with tumors of a higher grade, larger tumor size, and lymph node involvement compared to patients with a BMI in the healthy range [9, 25, 26, 28]. Long-term follow-up of breast cancer patients revealed that individuals who are obese also developed metastatic disease more rapidly after diagnosis with a higher frequency of distant recurrence than patients with a BMI in the healthy range (18.5–24.9 kg/m^2^) [25, 26, 28, 29]. Since obesity enhances progression, it has been hypothesized that increased tumor size and grade of the primary tumor at the time of diagnosis may contribute to the clinically observed increase in metastatic frequency [30, 31]. However, both clinical and pre-clinical studies have suggested that changes in the microenvironment of the tumor and surrounding breast tissue under conditions of obesity could promote expansion of metastasis-initiating cells, such as tumor cells that undergo a partial epithelial-to-mesenchymal transition (EMT), whereby tumor cells express mixed epithelial and mesenchymal genes [32–37]. Furthermore, systemic factors secreted from adipose tissue under conditions of obesity in the presence of a primary breast tumor could also enhance metastasis through recruitment of immune cell populations to distal metastatic sites [38–41]. Together, these studies suggest that obesity-induced changes in the tumor microenvironment may enhance tumor growth and aggressive characteristics leading to the clinically observed increased metastasis and worsened breast cancer survival in patients who are obese.

In addition to the increased risk of disease progression, breast cancer patients with BMI in the obese range are more likely to develop resistance to endocrine therapies and chemotherapies than women with BMI in the healthy range [25]. Postmenopausal women with a BMI greater than 30 kg/m^2^ and ERα^+^ breast cancers treated with aromatase inhibitors anastrozole or letrozole showed significantly decreased treatment efficacy compared to breast cancer patients with BMI in the healthy range [29, 42–44], yet no difference has been observed in the efficacy of the hormone therapy tamoxifen in breast cancer patients with differing BMI [43, 45]. While it has been hypothesized that the observed decrease in treatment efficacy of aromatase inhibitors in women with obesity could be due to an inadequate suppression of aromatase within adipose tissue [46], pre-clinical models have also suggested that alterations in the stroma within and surrounding breast tumors may also impact responsiveness [47, 48]. Obesity has also been associated with diminished efficacy of breast cancer chemotherapy [49–52], resulting in worse overall patient survival following treatment. Differences in response to chemotherapy may also be due in part to changes in the breast tumor microenvironment. Breast tumors from women with obesity have an elevated incidence of desmoplasia [53]. Desmoplastic tumors, which are characterized by increased numbers of cancer-associated fibroblasts (CAFs) within the stroma as well as deposition of fibrillar collagens, are associated with diminished survival in breast cancer patients [54–57]. Furthermore, desmoplasia in TNBC is associated with reduced relapse-free survival following chemotherapy [55, 56, 58, 59]. While other aspects of the breast tumor microenvironment have been recently reviewed [60], in this review, we will examine how obesity alters the cellular and extracellular structure of the tumor microenvironment to promote the growth of aggressive, treatment-resistant breast tumors.

## Cancer-associated fibroblasts and adipocytes

**Fig. 1:**
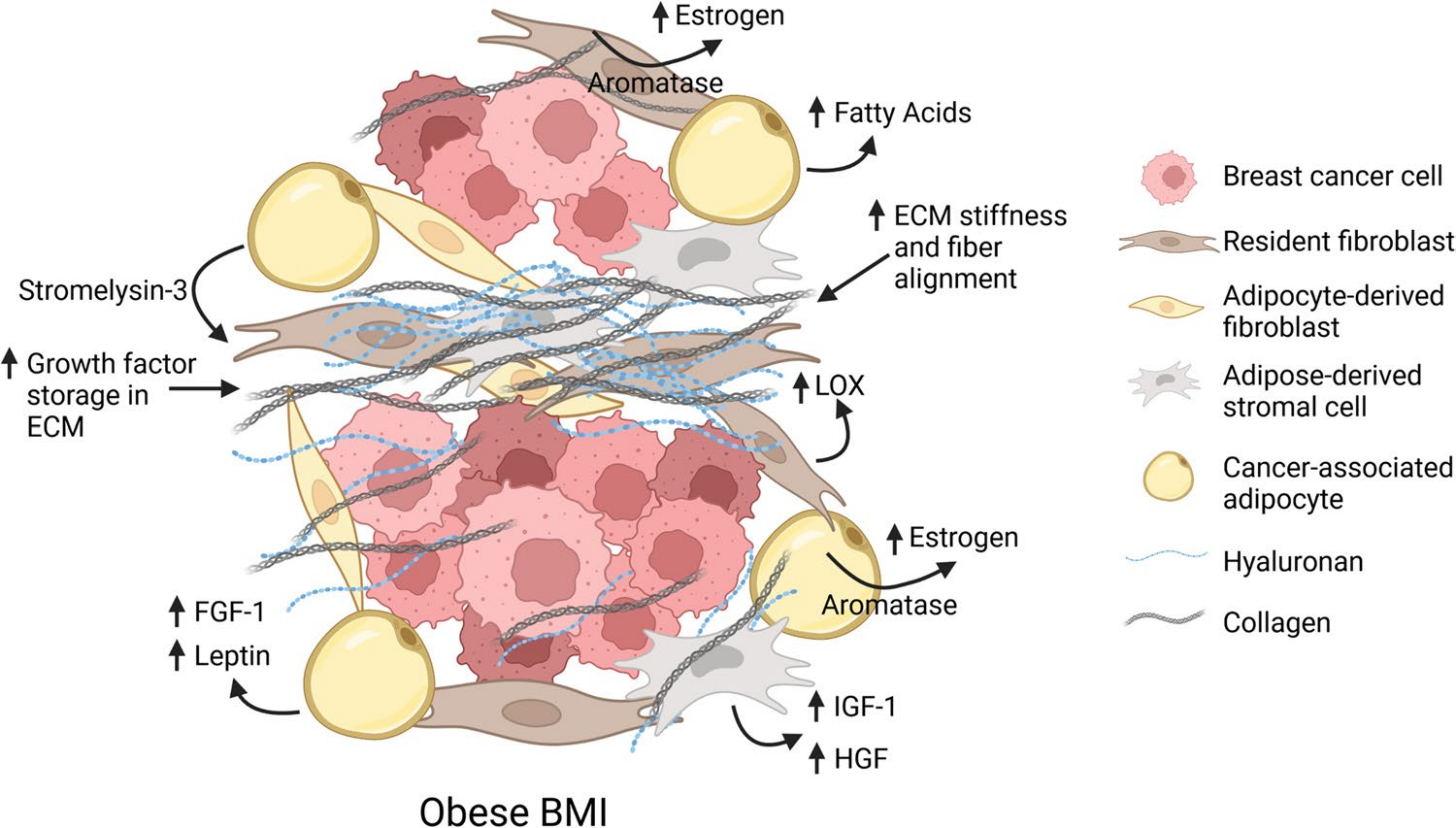
Breast tumors from patients with obesity demonstrate increased tumor desmoplasia. Under conditions of obesity, breast tumors have more aligned collagen within and surrounding the tumor as well as increased ECM components, including hyaluronan. CAFs from multiple sources, including resident fibroblasts, adipocyte-derived fibroblasts, and adipose-derived stromal cells, are increased in the tumor microenvironment in obesity and promote aggressive cancer cell growth through secretion of growth factors, elevation of ECM remodeling enzymes, and local production of estrogen through aromatase activity. Adipocytes within the tumor microenvironment alter tumor cell metabolism by enhancing fatty acids, secretion of growth factors and adipokines, and further elevating local levels of estrogen. Together, these cells contribute to chemotherapy resistance and accelerated breast tumor growth observed in patients with obesity

CAFs have diverse functions in the tumor microenvironment ranging from ECM deposition and remodeling to signaling interactions with cancer cells and surrounding immune cells. Although CAFs in breast tumors have been traditionally identified using the marker alpha-smooth muscle actin, CAFs are a heterogeneous population of cells that can be separated into subsets based on the expression of markers including fibroblast activation protein (FAP), fibroblast-specific protein-1 (FSP-1/S100A4), platelet-derived growth factor alpha (PDGFα) and PDGFβ [61–64]. CAFs have been suggested to originate from various sources, including resident fibroblasts, bone marrow-derived mesenchymal stem cells, pericytes, and tumor or endothelial cells that have undergone a mesenchymal transition [65, 66]. The impact of obesity on specific CAF populations within breast tumors is an emerging field. Obesity-induced changes in the function of stromal cells in the surrounding adipose tissue prior to tumor formation suggest that obesity could significantly alter the composition and function of CAF within the tumor stroma (Fig. 1).

Adipose tissue contains mesenchymal cells with the capacity for self-renewal and multipotent differentiation, including adipocytes, osteoblasts, and fibroblasts [67, 68]. Challenges exist in identifying and isolating adipose stem cells from other stromal cells present within adipose tissue, and studies examining adipose stem cells within the mammary gland frequently isolate a mixture of stromal cells, termed adipose-derived stromal cells. Adipose-derived stromal cells have the potential to differentiate into CAF within the tumor microenvironment, leading to mammary tumor progression [69], and obesity has been shown to promote adipose-derived stromal cell differentiation into myofibroblasts within tumors [53, 70, 71]. Adipose-derived stromal cells are expanded in obesity and can traffic from white adipose tissue into mammary tumors to promote growth [72]. Interestingly, obese breast cancer survivors have elevated circulating adipose-derived stromal cells compared to lean patients [73]. In pre-clinical models, increased numbers of these circulating cells play a role in distal metastases [72] through protection of circulating tumor cells from shear stress by enhancing cellular adhesions, preventing tumor cell apoptosis by activating cell survival pathways, and establishing metastatic niches at distal sites [74, 75].

Several co-culture experiments have demonstrated adipose-derived stromal cells from patients with obesity promote tumor cell migration, proliferation, and invasion [33, 34, 37, 76]. After exposure to adipose-derived stromal cells isolated from patients with obesity, breast cancer cells demonstrated increased ability to grow in suspension as tumorspheres, suggesting that the cancer cells had enhanced stem cell-like properties [34, 35, 37]. The increased invasive properties of tumor cells after exposure to adipose-derived stroma cells were abrogated by insulin-like growth factor-1 (IGF-1) neutralizing antibodies [37]. In a C(3)-Tag mouse model of basal breast cancer, smooth muscle actin-expressing myofibroblasts isolated from the mammary glands of obese mice produced elevated levels of hepatocyte growth factor (HGF), which significantly enhanced cancer cell proliferation and migration [70]. Adipose-derived stromal cells may also enhance the growth of ERα^+^ tumors. Adipose-derived stromal cells isolated from patients with obesity promoted secretion of leptin to regulate ERα^+^ MCF-7 tumor cell growth and aggressive tumor cell properties [33]. Silencing of leptin in adipose-derived stromal cells from patients with obesity led to reduced MCF-7 tumor cell expression of markers associated with EMT and reduced metastasis [33]. Additionally, adipose-derived stromal cells contribute to enhanced local levels of estrogen through expression of aromatase. Elevated levels of the inflammatory cytokine prostaglandin E2 in obesity led to downregulation of p53 in adipose-derived stromal cells resulting in increased aromatase expression and enhanced potential for ERα^+^ tumor formation [77]. Adipose-derived stromal cells may also contribute to treatment resistance within the breast tumor microenvironment. Adipose-derived stromal cells isolated from breast tissue from patients with obesity led to reduced sensitivity to the aromatase inhibitor, anastrozole, when co-cultured with MCF-7 cells in a 3D organotypic breast cancer model compared to adipose-derived stromal cells isolated from women with a healthy range BMI [47].

Breast adipose tissue exists adjacent to mammary ducts such that when breast tumors form, tumors are in close proximity to adipocytes. The proximity of cancer cells to adipocytes leads to the generation of fibroblast-like cells, termed adipocyte-derived fibroblasts, which may also be incorporated into the tumor stroma [78, 79]. Interestingly, adipocyte-derived fibroblasts do not express smooth muscle actin [78], which may contribute to the heterogeneity of markers observed in breast CAF. Loss of lipid droplets in adipocytes may be mediated in part through expression of stromelysin-3, which was found to be highly expressed in adipocytes at the invasive border of human breast tumors and diminished adipocyte differentiation in vitro [80]. It remains to be determined how obesity may alter the behavior of the adipocyte-derived fibroblasts within the tumor microenvironment.

Adipocytes have also been shown to enhance aggressive characteristics of tumor cells, which may contribute to tumor progression and metastasis. Co-culture of mature adipocytes with breast cancer cells promoted tumor cell proliferation [81–84], migration [81, 83, 85, 86], and invasion [83, 87]. When breast cancer cells were co-cultured with adipocytes isolated from patients with obesity, the capacity for tumor cell proliferation [32, 82], migration [32, 88], and invasion [88] was further increased. Numerous hypotheses have surfaced to delineate the mechanisms of how adipocytes isolated under obese conditions promote these phenotypes, including increased production of proinflammatory cytokines [87] and transcriptional regulation through microRNAs [83]. Balaban et al. demonstrated that breast cancer cells stimulated lipolysis of adipocytes isolated from obese patients in culture, leading cancer cells to accumulate adipocyte-derived fatty acids, which altered their cellular metabolism and induced proliferation and migration [32]. Obesity also regulates the expression of multiple adipokines through reduction of p16^INK4A^ in adipocytes [88]. Exposure of breast tumor cells to secretions of obesity-activated adipocytes led to expression of EMT markers [88]. Promotion of breast cancer cell EMT has been associated with increased expression of forkhead box C2, twist-related protein-1, and N-cadherin, along with decreased expression of E-cadherin, in tumor cells [89]. Through changes in adipocytes induced by obesity, mature adipocytes may promote tumor cell motility and selection for tumor cells with more aggressive characteristics. Furthermore, adipocytes may play a critical role in promotion of the growth of ERα^+^ tumors through elevated aromatase expression, as differentiated adipocytes have fivefold higher aromatase activity than adipose-derived stromal cells [90].

Adipocytes may also contribute to diminished efficacy of endocrine and chemotherapy treatments. Crosstalk between breast cancer cells and adipocytes led to suppression of the anti-proliferative effect of tamoxifen through adipocyte-induced changes in cancer cell gene expression [84, 91]. Expression of fibroblast growth factor receptor-1 (FGFR-1) on tumor cells is a known mediator of endocrine treatment resistance and is associated with poor breast cancer patient outcomes [92]. Adipocyte-mediated expression of FGF-1, the ligand for FGFR-1, is elevated under conditions of obesity in mice and humans [92], suggesting that adipocytes may contribute to endocrine resistance through upregulation of FGF-1. In addition to endocrine therapy resistance, adipocytes from patients with obesity have been shown to promote doxorubicin chemotherapy resistance [93]. When co-culturing obese adipocytes with breast cancer cells, adipocytes promoted the production of cytoplasmic vesicles by cancer cells, which sequestered doxorubicin, and were then expelled in the extracellular space [93], resulting in chemotherapy resistance in the tumor cells. Together, these studies suggest that both CAF within tumors and surrounding adipocytes may contribute both to the growth of aggressive breast tumors as well as resistance to treatment with multiple therapeutic agents.

## Extracellular matrix

The extracellular matrix (ECM) provides structural and mechanical support for breast tumors, influences the migration of tumor and immune cells through its physical properties [94, 95], and is composed of a complex meshwork of collagen fibers, glycoproteins, and proteoglycans [96]. Accumulating evidence suggests that the tumor ECM may promote resistance to chemotherapy, either by providing a protective barrier that reduces the concentration of anti-cancer drugs within tumors [97, 98] or by enhancing cancer cell survival [99]. Emerging evidence suggests that obesity significantly alters collagen alignment and ECM composition within breast tumors (Fig. 1). Collagen fibers, which are a major component of ECM, may impede tumor invasion by acting as a barrier against migration [100] or facilitate tumor cell invasion by acting as a directional scaffold for cellular movement based on the fiber orientation [101]. Tumor-associated collagen signatures (TACSs) of collagen fiber organization and alignment were identified in human breast tumors as a predictive marker for tumor progression and invasiveness [102]. TACS-3, which is a signature that reflects high local collagen density and perpendicular alignment of collagen fibers to the tumor boundary, has been shown to be an independent prognostic indicator for disease-free and disease-specific survival in breast cancer [103]. In breast tumors from women with obesity as well as mammary cell line-derived tumors in obese mice, collagen fibers were observed to be more aligned with each other within and surrounding the tumors compared to those of women with a healthy range BMI and lean mice [53, 104]. This collagen signature within breast tumors may also enhance metastasis, as breast tumor cells were observed migrating along aligned fibers and invading into the surrounding tissue [105]. Consistent with these observations, in a model utilizing transgenic mice with stabilized collagen fibers, ERα^+^ mammary tumors demonstrated significantly increased numbers of circulating tumor cells as well as increased metastasis [106]. Further studies are necessary to determine how targeting collagen alignment and organization could decrease metastasis in pre-clinical models of obesity-associated breast cancer.

In addition to collagen fiber alignment, mouse studies suggest that elevated stiffness in tumors contributes to cancer cell aggression and compromises treatment efficacy [107]. In vitro, ECM deposited by adipose-derived stromal cells isolated from the mammary glands of obese mice was stiffer than ECM deposited by adipose-derived stromal cells of lean mice [53]. Furthermore, differences in ECM production from adipose-derived stromal cells isolated from mammary tissue of obese mice promoted proliferation and migration of cultured preneoplastic mammary epithelial cells [53]. One possibility for differences in stiffness of the ECM may be due to increased expression of the collagen-crosslinking enzyme lysyl oxidase (LOX), which is enhanced in obese adipose tissue of both rats and humans [108, 109]. LOX overexpression in mouse mammary glands increased tissue stiffness, collagen deposition, and linearity of collagen fibers, while LOX inhibition resulted in less linear collagen fibers with decreased collagen deposition [107]. In a pre-clinical model of breast cancer, enhanced collagen expression and stabilization by LOX led to elevated tumor hypoxia, malignant signaling, and dysregulated angiogenesis [110]. In contrast, targeting LOX in tumors diminished chemotherapy resistance [110, 111]. Microdissected stromal cells from human breast tumors expressed the highest levels of LOX, which was significantly correlated with disease-specific mortality [112]. Together, these studies suggest that targeting of LOX may be a novel strategy to reduce ECM stiffness and potentially improve outcomes in breast cancer patients with obesity.

Obesity has also been shown to alter the composition of the ECM in multiple organs, including adipose tissue [113, 114]. Proteomics of the obese mammary gland and mammary tumors derived from multiple cancer cell lines identified a common signature of nine matrisome proteins, which were upregulated in both tumor and the obese mammary gland ECM, including collagen VI, collagen XII, fibronectin-1, laminin alpha subunit 5, vitronectin, tropoelastin, von Willebrand factor-1, galectin-1, and annexin A3 [115]. Increased deposition of collagen VI significantly enhanced the invasion of MDA-MB-231 and MDA-MB-468 TNBC cell lines [115]. Endotrophin, a cleavage product of collagen VI, promoted aggressive mammary tumors with high metastatic growth [116]. Within the ECM, hyaluronan is a linear polysaccharide that was considered to be an inert structural component; however, hyaluronan has been shown to activate kinase cascades in fibroblasts and bind to cell surface receptors such as CD44, which is expressed on the surface of aggressive breast cancer cells [117–119]. Hyaluronan expression was also observed to be significantly elevated in breast tissue from women with obesity and correlated with poor survival in breast cancer patients [120]. Another ECM component, heparanase, which is the sole mammalian endoglucuronidase that cleaves heparan sulfate in ECM, was identified as highly expressed in the ECM in obesity-associated human breast tumors [121]. Elevated heparanase may enhance local production of aromatase, the rate-limiting enzyme in estrogen biosynthesis through activation of inflammatory signaling in adipose tissue macrophages [121]. Changes in ECM composition surrounding breast tumors due to obesity could also impact the normal function of adipocytes and macrophages leading to further promotion of breast cancer growth [122, 123].

Obesity-induced changes in the ECM may also enhance tumor growth through release of growth factors that impact the tumor microenvironment [124]. In obese adipose tissue, transforming growth factor beta-1 (TGFβ1) concentrations are significantly enhanced [125–127], and TGFβ1 has been recognized as the most potent inducer of transformation of normal fibroblasts to CAF [128, 129]. TGFβ1 is produced as an inactive, latent form which complexes with latent TGFβ1 binding proteins as well as other matrix components including matricellular protein decorin within the ECM [130, 131]. In a high-fat diet model of obesity in mice, both decorin and TGFβ1 were significantly enhanced in the ECM surrounding mammary epithelial cells, and complexes of decorin and latent TGFβ1 were identified in ECM isolated from breast tissue from women with obesity [132]. Decorin is also significantly increased in the ECM of visceral and subcutaneous adipose tissue of obese patients [133]. Interestingly, loss of decorin within the ECM of ductal carcinoma in situ due to ECM remodeling is a marker for tumor progression and correlates with more aggressive disease [134, 135]. It is tempting to speculate that loss of decorin during tumor progression may increase TGFβ1 bioavailability in the tumor microenvironment. A number of growth factors, including IGF-1, FGF, and HGF, have also been found to associate with the ECM [136], and alterations in composition of the ECM due to obesity may alter the reservoir of growth factors stored in the ECM during tumor progression.

## Immune cells

**Fig. 2:**
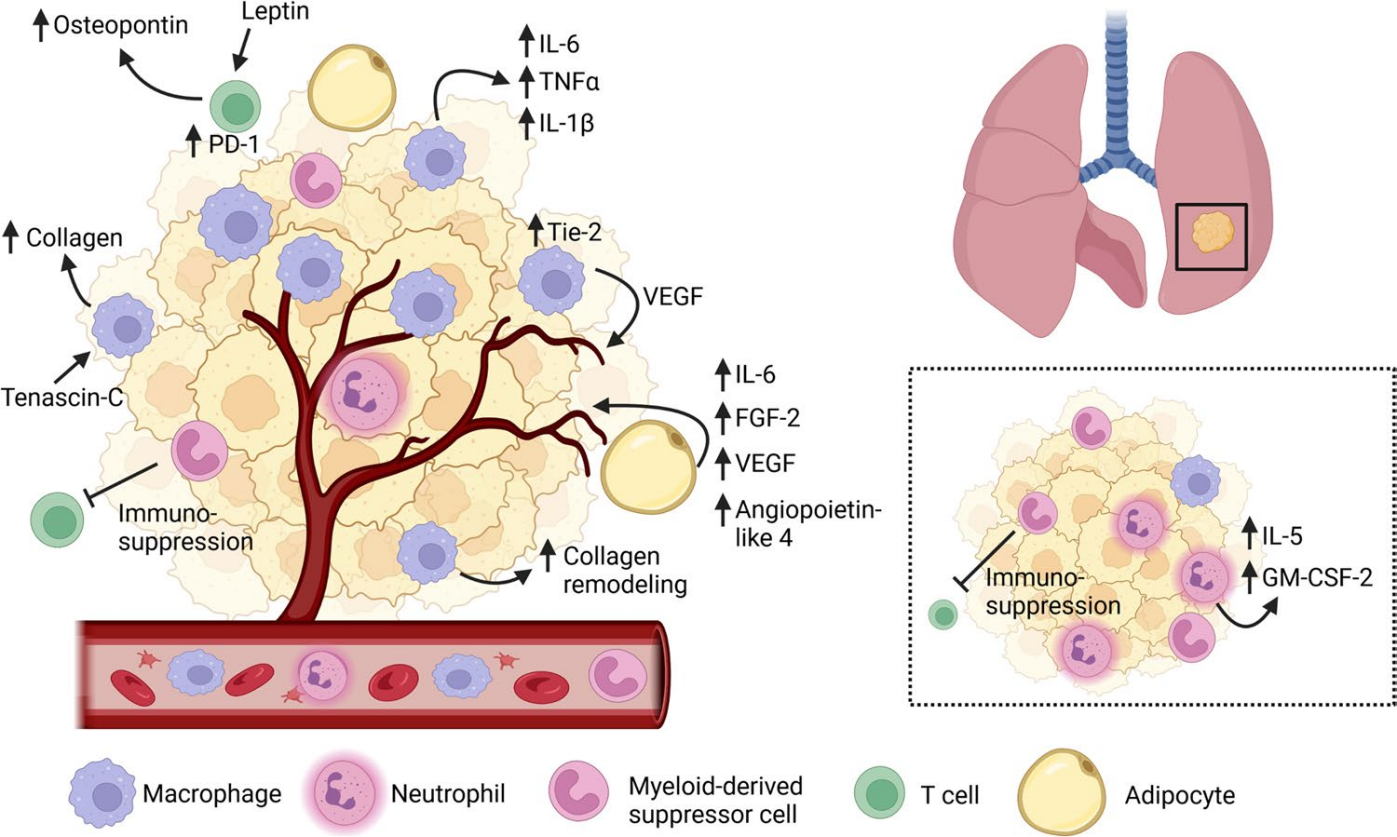
Obesity enhances myeloid-lineage cells within breast tumors and metastases. Chronic macrophage-driven inflammation within obese adipose tissue enhances circulating numbers of myeloid-lineage cells. Within breast tumors under conditions of obesity, TAMs play multiple roles including enhanced secretion of tumor-promoting cytokines, collagen deposition and remodeling, and promotion of angiogenesis. MDSCs have immunosuppressive functions within the tumor microenvironment and are increased in obesity. Adipocytes further enhance angiogenesis within breast tumors through secretion of VEGF and multiple cytokines and growth factors. Within surrounding adipose tissue, leptin increases PD-1 expression within CD8 + T cells and promotes an exhaustion phenotype. Within lung tissue of obese patients, macrophages, neutrophils, and MDSC are increased, leading to elevated extravasation of tumor cells and increased metastatic disease

Obesity alters the compliment and function of multiple different immune cell types within adipose tissue, and many of these changes may also be reflected in the tumor microenvironment, impacting the response of the tumor to therapy (Fig. 2). Tumor-associated macrophages (TAMs) have long been accepted as potent tumor promoters, acting through a variety of mechanisms to enhance tumor cell proliferation and invasion [137, 138]. Multiple therapeutic strategies impacting TAM survival or function have moved to phase I and phase II clinical trials [139, 140]. While pre-clinical studies have shown promise for therapeutic efficacy, significant questions remain regarding identification of patients that are most likely to respond to these therapies. Obesity is associated with chronic, macrophage-driven inflammation in breast adipose tissue, and the presence of macrophages surrounding necrotic adipocytes to form crown-like structures is considered to be a hallmark of obesity [141–143]. Macrophages forming crown-like structures secrete inflammatory cytokines such as tumor necrosis factor alpha (TNFα), interleukin-1β (IL-1β), and IL-6 and express marker the CD11c [142, 143]. Macrophages have been classified into M1 and M2 categories based on expression of cytokines in response to ex vivo stimulation [144]. Recent transcriptomic and proteomic studies have suggested that there is a more complex range of macrophage activation states than those captured using the M1/M2 classification system [145]. Macrophages in mammary adipose tissue of obese mice also appear to have unique metabolic activation due to the uptake of fatty acids released by adipocytes, leading to a distinct proinflammatory phenotype [146–148]. These macrophages, termed metabolically activated macrophages, have been shown to enhance markers of cancer stem-like cells in TNBC cell lines and have been identified within the breasts of women with obesity [146]. However, single cell sequencing of adipose tissue macrophages in obesity has revealed multiple distinct macrophage subtypes [149], and it is currently unclear whether these different subtypes of macrophages are present and have divergent functions in the breast tumors of women with obesity.

In mammary tumors of pre-clinical models, macrophages have been shown to promote cancer cells with cancer stem cell-like behavior, leading to increased metastasis and resistance to treatment with chemotherapy [150, 151]. Serum concentrations of TNFα are elevated in individuals with obesity [152], and macrophages are thought to be the major source of TNFα in obesity [153]. Treatment of TNBC cell lines with exogenous TNFα enhanced proliferation [154], which is hypothesized to contribute to rapid tumor growth in vivo. Depletion of macrophages using anti-F4/80 antibodies in obese tumor-bearing mice resulted in diminished tumor growth compared to tumors from obese mice treated with IgG antibodies [155]. Although tumor size was decreased in macrophage-depleted obese mice, cancer stem-like cells remained unchanged from those of control obese mice [155]. These data suggest that other factors altered by obesity may promote the accumulation of aggressive tumor cells, such as leptin secreted by adipocytes [35, 156]. Macrophages have also been associated with progression and early dissemination for metastasis in premalignant breast cancer lesions [157, 158]. However, depletion of macrophages early in tumor progression in obese mice in a p53-null model of mammary tumorigenesis led to increased DNA damage quantified in mammary epithelial cells [159], suggesting that macrophages may also serve numerous functions in the context of obesity. Further studies are necessary to determine how the function of TAM changes in the context of obesity in the tumor microenvironment.

Macrophages are directly influenced by the ECM, and changes in the ECM as a consequence of obesity could alter TAM function in the tumor microenvironment. When cultured on decellularized ECM isolated from adipose tissue of women with obesity, bone marrow-derived macrophages demonstrated a gene expression profile consistent with TAM expressing elevated transcripts of CD206 and arginase-1 compared to macrophages cultured on ECM from women with a healthy range BMI [123]. In the presence of IL-6 and collagen within the tumor microenvironment, a subset of FAP^+^ TAM expressed cytokines consistent with a wound healing response observed in macrophages isolated from wounded skin, and this gene expression profile was correlated with poor prognosis in breast cancer patients [160]. Given the elevated levels of collagen observed in breast tumors from women with obesity [53], it is possible that macrophages with this wound-healing signature could be similarly observed in breast tumors from women with obesity. In a pre-clinical model of breast cancer, the ECM component tenascin-C promoted an immunosuppressive TAM phenotype through toll-like receptor-4 (TLR4) signaling [161]. Expression of both tenascin-C and TLR4 was significantly increased in visceral fat of obese mice [162], which may suggest that these changes could be observed in breast adipose tissue surrounding tumors under conditions of obesity.

In addition to signaling changes in response to the ECM, macrophages may contribute to deposition of ECM in the tumor microenvironment under conditions of obesity. In obese adipose tissue, upregulation of the chemokine CCL2 led to chronic macrophage-driven inflammation [142, 163, 164]. Overexpression of CCL2 in the tumor microenvironment similarly led to increased TAM infiltration and collagen deposition [165, 166], which was abrogated by depletion of CD11b^+^ cells, including macrophages [166]. Furthermore, depletion of macrophages using clodronate-containing liposomes in mice bearing EO771 tumors resulted in altered collagen fibrillar microstructure as quantified using second harmonic generation imaging and immunofluorescence [167]. Together, these studies suggest that TAM can promote the organization and deposition of ECM within the tumor. In addition to exposure to tenascin-C altering the behavior of TAM to become more immunosuppressive [161], tenascin-C within the ECM also promoted the generation of TAM that exhibited increased synthesis and phosphorylation of collagen family members [168]. TAMs within tumors have also been observed to reorganize the collagen fibers favoring metastasis [167]. CCL5, another cytokine produced by adipocytes [169], has also been shown to enhance TAM recruitment into residual tumors in a conditional model of ErbB2 overexpression in mice, leading to increased collagen deposition within the tumor site and eventual recurrence of the tumor [170]. Together, these studies suggest that in obese patients, TAM may play a critical role in patients with obesity in promoting tumor recurrence and metastasis through remodeling the ECM in the tumor microenvironment.

Neutrophils have been shown to have both growth-promoting and growth inhibitory effects within the tumor microenvironment, which may be context-dependent [171]. In a retrospective study, a high circulating neutrophil-to-lymphocyte ratio in breast cancer patients with obesity was associated with a worse breast cancer-specific survival [172], which could reflect immunosuppressive functions. Within mammary tumors from mice fed a high-fat diet to induce obesity, neutrophil recruitment and tumor size were significantly increased compared to tumors from control mice [36], suggesting neutrophils may not only influence tumor initiation but also aid in progression under conditions of obesity. In culture, human breast cancer cell migration was stimulated by neutrophil secretions [173]. Although the role of neutrophils within the tumor microenvironment in obesity is not well understood, the role of neutrophils in the promotion of metastatic growth in obesity has been recently explored. Neutrophils have been implicated in obesity-associated lung metastasis by aiding in tumor cell seeding and growth through an IL-5 and granulocyte–macrophage-colony stimulating factor (GM-CSF)-dependent mechanism [38]. In a high-fat diet mouse model of obesity, neutralization of GM-CSF significantly decreased metastatic foci in lungs of obese tumor-bearing mice compared to lean mice [41]. This increased metastasis may occur in part through neutrophil-mediated impairment of vascular integrity under conditions of obesity, which enhanced cancer cell extravasation into the lung parenchyma [174]. Together, these results suggest that neutrophilia within the lungs induced by obesity plays a significant role in enhancing metastatic growth (Fig. 2).

Myeloid-derived suppressor cells (MDSCs) are a heterogeneous population of myeloid progenitor cells whose normal maturation into macrophages, dendritic cells, and neutrophils is impaired in tumors [175, 176]. Due to a lack of specific markers to definitively separate neutrophils from some types of MDSC, questions remain about the distinctive function of this group separate from neutrophils [171]. MDSCs are rarely found in adipose tissue in homeostatic conditions [177], but increase under conditions of obesity [178–180]. Although MDSCs represent a low percentage in the total number of immune cells in tumors, these cells appear to play a vital role in immune surveillance. MDSCs act to suppress inflammation, inhibit CD8^+^ T cell proliferation, and promote CD8^+^ T cell apoptosis [181]. In obese mice bearing either EO771 or PY8119 mammary tumors, granulocytic MDSCs were significantly increased through a CXCR1-mediated pathway and promoted the apoptosis of CD8^+^ T cells both in culture and in vivo [182]. Depletion of MDSC in mammary tumors of high-fat diet-fed mice significantly increased CD8^+^ T cell recruitment into tumors and decreased tumor volume [180]. Together, these data suggest that MDSC may promote tumor growth during obesity by impairing CD8^+^ T cell response. Tumor-infiltrating MDSC also demonstrated increased fatty acid uptake and activated fatty acid oxidation [183], which enhanced the immunosuppressive characteristics of the MDSC [184]. Obesity may significantly increase both the number and function of MDSC within the tumor microenvironment, which has implications both for tumor progression as well as potential immunotherapy strategies.

T cells respond specifically to antigens expressed by tumor cells, leading to antitumor immunity. Obesity is associated with reduced dendritic cell function and antigen presentation in mice [185], suggesting that obesity may diminish the responsiveness of the adaptive immune system to antigens from cancer cells. CD8^+^ T cells are the primary cells that eliminate tumor cells, and high CD8^+^ T cell infiltration into TNBC is associated with good clinical outcomes in breast cancer patients [186, 187]. However, when BMI is considered a variable, increased lymphocyte infiltration into tumors is not predictive for better survival in breast cancer patients with obesity [188]. In another study, obese breast cancer patients demonstrated lower total T cell infiltration within tumors prior to treatment compared to women with a healthy range BMI [189]. Decreased T cell infiltration in mammary tumors has also been observed in pre-clinical models [155, 182, 190, 191], which may suggest reduced immunosurveillance in tumors under conditions of obesity. In a pre-clinical model, treatment of mammary tumor-bearing mice with 27-hydroxycholesterol resulted in fewer cytotoxic CD8^+^ T lymphocytes within the tumor microenvironment [192]. These results suggest that elevated circulating levels of cholesterol could play a role in diminished CD8^+^ T cell recruitment in obesity and are consistent with the observation that use of statins to decrease circulating cholesterol levels leads to diminished cancer mortality [193]. Obesity has also been associated with loss of T cell diversity and dysfunctional T cell responses to viruses and cancer [194, 195]. One dysfunctional T cell state is exhaustion, which is characterized by distinct epigenetic and transcriptional phenotypes, loss of effector functions, and prolonged and increased expression of inhibitory markers, such as programmed cell death protein-1 (PD-1). T cell exhaustion is thought to occur due to chronic antigen stimulation [196, 197]. While CD8^+^ effector T cells are recruited to adipose tissue during the early stages of obesity [198, 199], recent studies have suggested that these cells also express PD-1 and have diminished responses in the absence of tumors in obese mice [200]. These studies suggest that CD8^+^ T cell dysfunction may occur prior to tumor formation in obesity. Within tumors of obese mice, excess production of leptin led to an increase in fatty acid oxidation in CD8^+^ T cells and reduced interferon-gamma expression [201], which is an indicator of T cell exhaustion [202]. This data is supported by a study from Kado et al. which identified that obesity promoted a switch from non-exhausted PD-1 negative CD8^+^ T cells to exhausted PD-1^+^ CD8^+^ T cells in mammary tumors from obese mice [203]. In addition, the exhausted PD-1^+^ CD8^+^ T cells demonstrated increased expression of osteopontin [203], which has previously been implicated in tumor progression by regulating multiple pathways [204]. Together, these studies suggest that obesity diminishes both the number of CD8^+^ T cells within the tumor microenvironment and inhibits their function.

In addition to CD8^+^ T cells, obesity has been shown to impact other T cell subtypes which may promote breast cancer growth. Within obese adipose tissue, increased expression of TNFα has been shown to reduce CD4^+^ T cell function [205], suggesting that obesity may also inhibit the function of CD4^+^ T cells early during breast tumorigenesis. Natural killer (NK) cells, which are a critical part of the innate immune system, are impaired in patients with obesity through elevated circulating levels of leptin [206–208]. A prospective study demonstrated that dysfunctional NK cells were associated with an increased breast cancer incidence [209]. Furthermore, lipid accumulation in NK cells isolated from individuals with obesity was associated with the loss of NK cell cytotoxicity against tumor cells which could be restored through metabolic reprogramming [210], suggesting that changes in lipid metabolism associated with obesity may also alter the function of NK cells. Another T cell subtype which has innate immune cell function is γδ^+^ T cells. IL-17-secreting γδ^+^ T cells have been shown to promote primary tumor growth and metastasis, both in mice and in humans [211]. Pro-tumoral IL-17^+^ γδ^+^ T cells selectively showed high lipid uptake and intracellular lipid storage and were expanded in mammary tissue and tumors of obese mice [212]. Together, these studies suggest that obesity-induced changes in multiple immune cell populations enhance tumor growth and progression.

## Endothelium

Expansion of adipose tissue is coordinated with its vascularization, and adipocytes secrete multiple pro-angiogenic factors including vascular endothelial growth factor (VEGF), FGF-2, IL-6, CCL2, and leptin to coordinate this process [213]. Increased levels of these pro-angiogenic factors are found in circulation in women, rats and mice associated with obesity [214–216], which may cooperatively enhance angiogenesis within breast tumors. Increased microvascular density within breast cancer is correlated with progression, metastasis, and reduced overall survival in breast cancer patients [217, 218]. Elevated microvascular density has also been observed in obesity and associated with rapid tumor growth in canine mammary cancer [219] as well as multiple pre-clinical models of obesity and breast cancer. In a mouse model of postmenopausal obesity, EO771 tumors in the mammary glands of obese ovariectomized mice demonstrated significantly increased blood vessel density mediated by elevated levels of VEGF secreted by surrounding adipocytes [220]. Similarly, MMTV-PyMT transgenic mice fed a high-fat diet developed tumors with increased microvascular density and recruitment of macrophages through elevated levels of CCL2 [221]. In an isocaloric diet model, mice fed a diet high in cholesterol demonstrated the highest growth rate of MDA-MB-231 tumors, which had the highest microvascular density [222], suggesting that elevated cholesterol levels could play a role in increased angiogenesis in obese individuals. In culture, leptin and IL-6 produced by obese adipocytes induced activation, proliferation, and migration of endothelial cells through upregulation of VEGF and VEGFR-2 [91, 223], suggesting that adipocytes secrete multiple factors that could enhance angiogenesis within the tumor microenvironment. While increased blood vessel density and angiogenesis may suggest that individuals with obesity could be candidates for anti-angiogenic therapeutics, breast cancer patients with obesity treated with anti-VEGF antibodies were less sensitive to anti-VEGF treatment due to increased systemic concentrations of IL-6 and FGF-2, which compensated for VEGF inhibition [224]. Similarly, treatment of tumor-bearing obese mice with anti-mouse VEGF antibodies demonstrated reduced sensitivity to treatment, which was determined to be due to elevated body weight rather than diet [224]. Together, these studies suggest that anti-angiogenesis therapies in obese breast cancer patients may need to target multiple pathways due to the upregulation of multiple angiogenic factors secreted by obese adipocytes.

Macrophages recruited to the obese tumor microenvironment may further stimulate tumor angiogenesis by providing soluble growth factors, matrix remodeling enzymes, and other bioactive molecules that promote the growth and migration of endothelial cells. Macrophages have been shown to secrete multiple pro-angiogenic factors including VEGF, TNFα, GM-CSF, and IL-6 [225]. Macrophages isolated from the mammary tumor microenvironment of obese mice demonstrated elevated tie-2 expression [226], which may suggest that obesity enriches for a subpopulation of macrophages that are known to promote angiogenesis. In a humanized mouse model, elevated CCL2 within the mammary tumor microenvironment enhanced macrophage recruitment, and depletion of macrophages led to decreased blood vessel density and diminished tumor growth [227]. Crosstalk between macrophages and adipocytes may also contribute to the increased angiogenesis observed in obese breast cancer. Co-culture of breast adipocytes with THP-1 macrophages stimulated expression of VEGF-A in macrophages [228]. In obese mice, IL-1β produced by macrophages enhanced expression of VEGF as well as angiopoietin-like 4 from adipocytes surrounding mammary tumors [229, 230]. Transplant of EO771 tumor cells into the mammary glands of angiopoietin-like 4-null mice resulted in significantly reduced tumor growth with limited blood vessel density [230]. Macrophages also express the receptor for leptin [231], and leptin secretion by adipocytes may enhance the angiogenic factors secreted by macrophages. Given the number of angiogenic factors that are produced by macrophages under conditions of obesity, therapeutically targeting macrophages rather than individual angiogenic factors may lead to improved anti-angiogenic responses.

## Opportunities to improve therapeutic outcomes

The effects of weight loss on the risk for breast cancer recurrence are currently under investigation in long-term, clinical trials [232]; however, little is known about how weight loss may impact the tumor microenvironment and response to therapies. Mammary glands of obese mice that underwent weight loss demonstrated global epigenetic programming more similar to adipose tissue from obese mice than lean controls [233], suggesting that weight loss is insufficient to revert hypermethylation of genes in the mammary gland during the weight loss period examined. In adipose-derived stromal cells isolated from formerly obese mice, some changes in proliferation and viability were improved compared to those isolated from obese mice; however, impaired oxidative respiration was still observed [234]. Together, these data suggest that some obesity-induced changes in adipose-derived stromal cells could be reversed with weight loss, but not others, which could impact stromal cells that contribute to the formation of CAF. In obese mice, an 8-week weight loss period resulted in significantly decreased accumulation of macrophages in subcutaneous adipose tissue, suggesting that caloric restriction may resolve some inflammation associated with obesity [235]. Similarly, weight reduction significantly reduced the number of crown-like structures present within the mammary glands of formerly obese mice; however, expression of inflammatory molecules, including IL-6, TNF-α, and IL-1β, remained significantly elevated in the mammary glands of weight loss-induced mice relative to lean mice [236]. Elevated expression of inflammatory mediators within the mammary gland could still promote angiogenesis in the tumor microenvironment. More studies are necessary to understand how weight loss may impact the function of cell populations within the tumor microenvironment that contribute to tumor aggressiveness and therapeutic resistance. Weight loss leads to decreased recruitment of macrophages into obese adipose tissue concurrent with reduced circulating myeloid-lineage cells systemically. In a pre-clinical model, formerly obese mice demonstrated significantly reduced lung neutrophilia, resulting in reduced mammary cancer metastatic foci within the lungs compared to obese mice [38]. Additional studies could shed light on how weight loss impacts breast cancer metastases at other sites, including bone, brain, and liver.

In addition to weight loss, other therapeutic strategies targeting the tumor microenvironment may be beneficial for obese breast cancer patients. Metformin is a commonly utilized drug in the treatment of type 2 diabetes and has limited toxicity when used in combination with other breast cancer therapies [237, 238]. In mammary tumor-bearing obese mice, metformin treatment reduced obesity-associated tumor growth and was associated with a marked decrease in angiogenesis within the tumor microenvironment [229]. Similarly, treatment of obese/diabetic mice with metformin during EO771 tumor growth resulted in reduced tumor growth rates with less aligned collagen, decreased vascularity, reduced numbers of CD206^+^ TAM, and diminished pulmonary metastasis [239]. Furthermore, treatment of ovariectomized rats with metformin decreased the growth of ERα^+^ mammary tumors and diminished aromatase expression in CD68^+^ macrophages [240]. Together, these results suggest that metformin may have therapeutic effects on multiple cell types within the breast tumor microenvironment. Multiple ongoing clinical trials are currently being conducted to assess the efficacy of metformin as an adjuvant therapy for early stage, invasive, and metastatic breast cancer. In the recent MA32-randomized breast cancer trial, patients with breast cancer treated with metformin resulted in mild weight reduction and improvement in metabolic parameters compared to patients receiving placebo [241]. Further analysis of the trial is ongoing to assess breast cancer outcomes and potential impact on the tumor microenvironment.

Another potential target for therapy is the renin-angiotensin axis. Although most characterized for regulation of blood pressure, the renin-angiotensin system has also been recognized for its pathological role in obesity and breast cancer [242]. Angiotensin expression is enhanced in obese adipocytes and has been linked to elevated inflammation and metabolic disturbance [242]. In a mouse model of breast cancer, tumor desmoplasia was significantly reduced and chemotherapy outcome was improved after the administration of angiotensin receptor blockers conjugated to nanopolymers that degraded selectively in the tumor microenvironment [243]. Together, these results suggest that targeting this pathway may improve chemotherapeutic efficacy for women with obesity. Additionally, therapeutics targeting adipose-derived stromal cells are being developed in pre-clinical models. Novel killer peptides, which target an adipose-derived stromal cell-binding domain of decorin, demonstrate dose-dependent cytotoxic specificity for adipose-derived stromal cells [244, 245]. Su et al. demonstrated that when prostate cancer cells were xenografted into obese and lean mice, treatment with the killer peptide D-WAT significantly reduced prostate cancer cell invasion and tumor growth in obese mice compared to vehicle-treated obese and lean mice [246]. These results suggest that targeted reduction of adipose-derived stromal cells in obese mice reduces aggressive characteristics of tumors, which may improve response to clinical treatments.

Multiple strategies have also emerged to therapeutically target TAM. As CCL2 upregulation in obesity promotes macrophage recruitment into adipose tissue [142, 163, 164], inhibitors of CCL2 have been developed to reduce TAM within the tumor microenvironment and at sites of metastasis. In mice bearing late-stage MMTV-PyMT mammary tumors with spontaneous metastasis, treatment with CCL2 inhibitors reduced metastasis through diminished recruitment of metastasis-associated macrophages [247]. However, cessation of CCL2 therapy led to increased and rapid metastasis in multiple pre-clinical mammary tumor models through VEGF-mediated angiogenesis [248]. Elevated circulating levels of CCL2 following treatment could potentially be a significant problem in breast cancer patients with obesity. Colony-stimulating factor-1 (CSF-1) inhibitors have also been developed for clinical use and act to both diminish TAM recruitment as well as promote macrophage apoptosis [249]. The efficacy of this approach may be limited by CAF in the tumor microenvironment, which promoted an increase in MDSC within tumors in response to treatment with CSF-1R inhibitors [250]. Since MDSCs are increased under conditions of obesity [178–180], the efficacy of CSF-1R inhibitors should be examined in obese pre-clinical models. Other strategies to alter the function of macrophages may prove beneficial to breast cancer patients with obesity. In a model of pancreatic adenocarcinoma, PI3Kγ inhibition led to improved CD8^+^ T cell responses, diminished tumor cell metastasis, and reduced desmoplasia [251]. However, PI3Kγ has been shown to regulate lipid metabolism in adipose tissue macrophages, and downregulation of PI3K signaling may lead to elevated macrophage lipid burden and death and further contribute to metabolic pathogenesis in obesity [252].

Although obesity appears to have detrimental effects on the adaptive immune system, clinical studies have surprisingly revealed increased responses to immunotherapies in obese cancer patients. Immune checkpoint inhibitors, particularly agents targeting PD-1 or its ligand PD-L1, have been approved for treatment of various cancers [253]. For breast cancer patients, treatment of metastatic, TNBC with PD-1 inhibitors is efficacious in approximately 18% of patients [254]. The mechanisms underlying these limited treatment responses are not clear, and biomarkers identifying patients that may respond to these therapies have not been delineated. In a cohort of cancer patients treated with anti-PD-L1 therapy, a larger percentage of patients with a BMI greater than 30 kg/m^2^ had increased progression-free survival and overall survival compared to patients with a BMI less than 30 kg/m^2^ [255]. This trend for increased efficacy of immunotherapy observed in multiple types of solid tumors could also apply to TNBC patients with obesity [256], and recent studies have demonstrated improved immunotherapy responses in pre-clinical models of breast cancer in obesity [257]. The chronic inflammation observed in the mammary gland and tumor microenvironment as a consequence of obesity and resulting T cell exhaustion may lead to a microenvironment with greater responsiveness to treatment with checkpoint inhibitors. However, there is also evidence that patients with obesity have an increased incidence of immune-related adverse events when treated with PD-1 therapy that result in discontinuation of treatment [258]. Further clinical trials are necessary to determine how obesity impacts response to immunotherapy in breast cancer. Pre-clinical studies in this area will help guide new therapeutics for targeting the immune response to improve cancer outcomes for patients with obesity.

## Conclusions

The tumor microenvironment is complex, with interactions among different cell types supporting the growth and malignancy of tumor cells. Obesity alters the function of multiple cell types within the tumor microenvironment, leading to the growth of breast tumors with increased propensity for therapy resistance and recurrence. While we are beginning to uncover how obesity shapes individual cell types within the tumor microenvironment, there is much to be discovered in how these cell types interact and are further modified by the hormonal changes of menopause. Furthermore, breast cancer is made up of multiple subtypes, each with distinct differences in the cell populations that make up the tumor microenvironment. Continued refinement of pre-clinical models to examine how obesity alters the function of stromal cells within different breast cancer subtypes is necessary to improve treatment responses for patients with obesity and identify potential targets for the development of new therapeutic interventions.
